# Synaptic Protein Phosphorylation Networks Are Associated With Electroacupuncture-Induced Circadian Control in the Suprachiasmatic Nucleus

**DOI:** 10.3389/fgene.2021.762557

**Published:** 2021-12-16

**Authors:** Xiaoxiao Lu, Minjie Zhou, Nannan Liu, Chengshun Zhang, Zhengyu Zhao, Dingjun Cai

**Affiliations:** Acupuncture and Tuina School, Chengdu University of Traditional Chinese Medicine, Chengdu, China

**Keywords:** circadian rhythm, phosphorylation, mass spectrometry, suprachiasmatic nucleus, electroacupuncture

## Abstract

Phosphorylation is one of the most important posttranslational modifications and regulates the physiological process. While recent studies highlight a major role of phosphorylation in the regulation of sleep–wake cycles to a lesser extent, the phosphoproteome in the suprachiasmatic nucleus (SCN) is not well-understood. Herein, we reported that the EA treatment elicits partial reparation of circadian rhythmicity when mice were exposure to constant darkness for long time. We investigated the effects of EA on circadian rhythms in constant darkness between EA stimulation and free-running control. Next, mass spectrometry–based phosphoproteome was utilized to explore the molecular characteristics of EA-induced phosphorylation modification in the SCN. A total of 6,192 distinct phosphosites on 2,488 proteins were quantified. Functional annotation analysis and protein–protein interaction networks demonstrated the most significant enriched phosphor-proteins and phosphosites involved in postsynapse and glutamatergic synapse. The current data indicated that most of the altered molecules are structural proteins. The target proteins, NMDAR and CAMK2, were selected for verification, consistent with the results of LC–MS/MS. These findings revealed a complete profile of phosphorylation modification in response to EA.

## Introduction

The suprachiasmatic nucleus (SCN) is a mammalian master clock that regulates various circadian rhythms, such as physiology and behavior, to adapt the internal environment to changes in the external environment. The SCN can be entrained by zeitgeber to regulate the biological rhythms. Several studies have been conducted to examine the proteomics of the SCN to elucidate the circadian rhythm (Deery et al., 2009; Chiang et al., 2014). The posttranslational modifications (PTMs) can alter the activity of proteins involved in cell signaling. Phosphorylation is a major PTM (Robles et al., 2017). Two recent reports highlighted the role of phosphorylation in the regulation of sleep–wake cycles by allowing prompt modulation of protein activity (Wang et al., 2018; Brüning et al., 2019). Despite extensive research on phosphorylation in the liver, little is known about its role in the regulation of the SCN circadian rhythm.

Acupuncture is an ancient practice to treat sleep disturbances (Huang et al., 2018). Laser acupuncture can help patients with circadian rhythm disorders (Wu et al., 2009). Reportedly, electroacupuncture (EA) regulates the expression of circadian rhythm genes (*Per1* and *Per2*) in SCN under the pathological condition of morphine tolerance (Hou et al., 2018). It has been shown acupuncture treatment could improve the circadian rhythm of blood pressure (Kim et al., 2012; Yang et al., 2016). EA also decreases phosphorylation of the N-methyl-D-aspartate (NMDA) receptor subunit GluN1 of the spinal cord in a persistent pain model (Zhang et al., 2014). Traditional Chinese medicine has emphasized the circadian rhythm of diseases for a millennium. This information was used to arrange the acupuncture treatment at appropriate hours of the day (Samuels 2000). A previous study described evidence that acupuncture has different effects on fibroblast cytoskeleton remodeling at different time points (Liu et al., 2020). It could be a critical first step in chronotherapy, and analyzing the impact of acupuncture on phase synchronization within the circadian network would provide the chronotherapy tool. Therefore, in this study, we investigated the effect of EA on circadian rhythm at different circadian time points. Also, multiplex tandem mass tag (TMT)–labeling coupled with liquid chromatography–mass spectrometry (LC-MS) was used to study the rhythmic regulatory effects of EA at different time points and the mechanism of EA-associated SCN protein phosphorylation. To the best of our knowledge, this is the first phosphoproteomic study of the SCN to reveal circadian control of EA that highlights the EA contributions to phosphorylation in SCN on a system level.

## Materials and Methods

### Animals and Housing

Eight-week-old male Balb/c mice were used in this study because female mice have periodical estrum cycles that show irregular activity levels. Each animal was housed in a standard cage (40 × 22 × 20 cm) with a running wheel (10 cm diameter). The wheel running data of each mouse were recorded throughout the process using ClockLab analysis software (version 3.208, Actimetrics).

### Electroacupuncture Treatment

**FIGURE 1:**
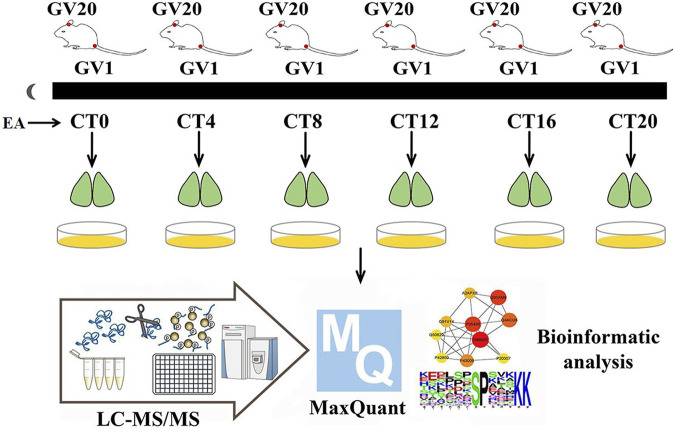
Experimental workflow. On day 10 of constant darkness, the EA group mice were treated by EA at CT0, CT4, CT8, CT12, CT16, and CT20, respectively. The phase shift value was tested by ClockLab. Two hours post-EA stimulation, the SCN tissues were collected and iced in a quickly. Then mass spectrum data were collected, and MaxQuant was used for protein estimation.

The EA group mice received EA at the corresponding circadian time points (CT0, CT4, CT8, CT12, CT16, and CT20). Acupoints GV1 (Changqiang) and GV20 (Baihui) were selected in this study (Figure 1). The research showed that electroacupuncture at GV1 and GV20 can restore the rats’ circadian rhythm of heart rate back to normal quickly, caused by a reversed light/dark cycle (Xue et al., 2010). GV-1 is an acupuncture point located at the midpoint of the line between the tailbone and the anus (Cerreta et al., 2018). GV20 is located above the apex auriculate, in the midline of the head. After piercing, the needles were connected to the Hans acupoint nerve stimulator that sent an electric current frequency of 2/15 Hz, sparse-dense wave, and a current of 0.5 mA for 15 min. As described by Zhang et al (Zhang et al., 2020), EA mice immobilized by two Velcro brand hooks and loop fasteners; additional tapes fixed the animal to a wooden block only for the duration of EA, and the control group also received immobilization at the same period. Similar to previous proteomics studies on acupuncture (Xu et al., 2020), since there was not a disease model group, only the control group and EA group were set up. We want to investigate if EA entrainment only once is sufficient to trigger phase shift compared to the control group under constant dark background.

### Experimental Schedule

Balb/c mice were housed in an environment with free access to get food and water and 12-h/12-h light–dark cycle (7 a.m. lights on) for 10 days, constant temperature (23 ± 1°C), and humidity. A total of 72 mice were randomly divided into six time point groups (CT0, CT4, CT8, CT12, CT16, and CT20); each CT group was divided into EA and control (non-EA) groups. After 10 days of light–dark acclimatization, the animals were kept in constant darkness (dark–dark, DD) for 10 days. After all the animals were acclimatized for 10 days, those not synchronized in the light–dark cycle were eliminated. The phasing of the EA group was examined according to the Aschoff type I protocol (Aschoff 1984). All manipulations of DD mice were performed under red light (5 Lux). On day 10 of constant darkness, the EA group was treated with EA at CT0, CT4, CT8, CT12, CT16, and CT20, respectively, with six mice for each time point. ClockLab software was used to calculate the predicted activity’s onset time; the actual activity onset time was day 10, following which the CT time was determined. The predicted initial activity time of each animal was determined by the linear regression equation based on the actual activity onset time. Then the phase shift value of each animal was calculated. Similar to previous studies, mice were killed by acute cervical dislocation after 2 h post-EA (Sundquist and Nisenbaum 2005; Chen et al., 2017). The brains were dissected and cut into 800-µm-thick coronal sections containing the SCN in cooled oxygenated media. The SCN region was isolated using microsurgery forceps and knives according to the Allen Mouse Brain Atlas (Chiang et al., 2014). TMT-labeled phosphorylation was applied. The protein extracts from the three SCN were mixed in equivalent amounts for two samples per time point for each time point. A total number of 24 samples were used for peptide extraction.

### Protein Extraction

Mouse SCN tissues were quickly frozen in liquid nitrogen. The sample was ground into a cell powder and resuspended in four volumes of lysis buffer (8 M urea, 1% phosphorylase inhibitor cocktails, 1% protease inhibitor), followed by sonication three times on ice. The supernatant was collected by centrifugation at 12,000 *g*, 4°C for 10 min, and the protein concentration was determined using the BCA kit.

### Phosphoproteome Sample Preparation

The protein solution was reduced with 5 mM dithiothreitol at 56°C for 30 min, followed by the addition of iodoacetamide to a final concentration of 11 mM and incubation in the dark for 15 min. Subsequently, the protein was digested with trypsin at 1:50 and 1:100, respectively (Huang et al., 2020; Feb 1). The peptide was solubilized in 0.5 M TEAB and labeled according to the TMT kit protocol method. One unit of the TMT labeling reagent was dissolved in acetonitrile, mixed with the peptide, and incubated at room temperature for 2 h. The Strata X C18 SPE column (Phenomenex) was used for desalting and lyophilizing peptides in vacuo. The peptide mixtures were solubilized in the buffer (50% acetonitrile/6% trifluoroacetic acid), and the supernatant was transferred to the IMAC microspheres and mixed with gentle shaking by a shaker. The buffer of 50% acetonitrile/6% trifluoroacetic acid and 30% acetonitrile/0.1% trifluoroacetic acid was used sequentially to wash the mixtures three times. Finally, the phosphopeptide was eluted in 10% NH_4_OH, frozen, drained, and desalted. The reagents used in the experiment were HPLC grade.

### LC-MS/MS Analysis

The peptides were solubilized in 0.1% formic acid solution and separated by the EASY-nLC 1000 UPLC system (Thermo Fisher Scientific) at a flow rate of 700 nL/min. Buffer A was composed of 0.1% formic acid and 2% acetonitrile, and buffer B was composed of 0.1% formic acid and 90% acetonitrile. The gradient was set at 4–80% for 60 min. The peptides were introduced into the NSI source for ionization and analyzed by Q-Exactive^TM^ Plus (Thermo Fisher Scientific) mass spectrometry. The voltage was set to 2.0 kV. Both the peptide and its secondary fragments were detected using Orbitrap. The range for the mass spectrometer was 350–1800 m/z, and the secondary scan resolution was set to 35,000. The top 10 peptide ions were isolated with the highest signal intensity to enter a high-energy collisional dissociation (automatic gain control 5E4, signal threshold 5E3, maximum injection time 200 m, 28% collision energy, and dynamic exclusion time 15 s).

### Database Search

Raw MS files were processed using MaxQuant software (version 1.5.2.8) on the SwissProt mouse database of 16,964 sequences with a false discovery rate (FDR). The FDR of protein identification and peptide-spectrum matches was set to <1%. The number of trypsin/P missing cleavages was set to 2, and seven amino acid residues were defined as the minimum length of the peptide. Cysteine alkylation is set as fixed modification, and variable modification is the oxidation of methionine, acetylation of the N-terminus of the protein, and the phosphorylation of serine, threonine, and tyrosine. Data are available via ProteomeXchange with identifier ProteomeXchange: PXD029450.

### QC Validation of MS Data

Quality control results: The mass error of the center shaft was 0 and was found to be <10 ppm, indicating that the quality error was within the normal range. The length of the peptide was controlled at 8–20 amino acid residues, according to the law of trypsin digestion (Supplementary Material Figure S1).Supplementary Figure S1 Quality of mass spectrometry data: (A) Mass error distribution of all identified peptides. (B) Length distribution of peptides.

### Bioinformatics and Statistical Analysis

At least two independent biological replicates were run for each experiment. UniProt ID was used for annotation analysis. Metascape protein function and pathway annotation were applied through Gene Ontology (GO), Kyoto Encyclopedia of Genes and Genomes (KEGG), and Reactome and WikiPathways (Zhou et al., 2019). Enrichment analysis was performed using Fisher’s exact test. Protein–protein interaction (PPI) networks were generated from the STRING database (Szklarczyk et al., 2019) and visualized with Cytoscape 3.8.0. (Shannon et al., 2003).

### Immunohistochemistry (IHC)

IHC was performed as described previously (Mori and Cardiff 2016). Tissues are loaded into embedding cassettes. Then the paraffin sections were deparaffinized and rehydrated. The antigen retrieval was performed by immersing the slides in sodium citrate buffer (10 mM, pH = 6.0) and heating in a microwave oven for 10 min. After cooling, the slides were washed three times with phosphate-buffered saline (PBS) for 5 min. Endogenous peroxidase was blocked using 3% hydrogen peroxide at room temperature for 10 min, followed by washing with PBS. Then the slides were blocked with normal goat serum at room temperature for 20 min. Primary antibodies were added to each section and incubated at 4°C overnight, followed by incubation with secondary antibody at 37°C for 30 min. The tissue slide was then immersed in the DAB solution and rinsed with distilled water. IHC antibodies were obtained from Abcam. Image data were analyzed using Indica Labs (United States). All statistical analyses were performed using SPSS software 23.0 (IBM Corporation, United States). The differences in immunopositivity and staining were assessed using Student’s *t*-test. All IHC tests were carried out blind.

## Results

### Phase Shifts

**FIGURE 2:**
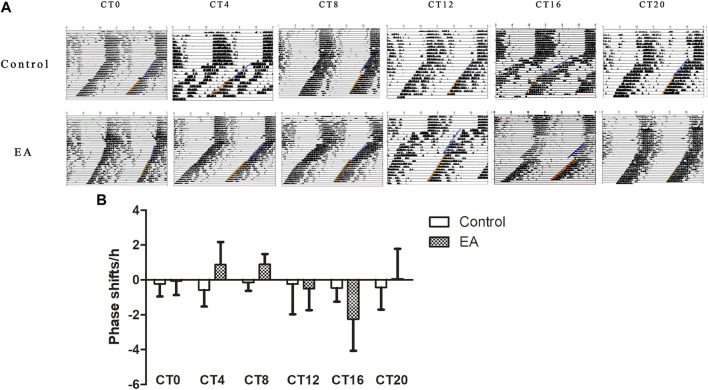
On day 10 of constant darkness, the EA group mice received electroacupuncture at CT0, CT4, CT8, CT12, CT16, and CT20, respectively. Two acupoints, GV1 (Changqiang) and GV20 (Baihui), were adopted using standard acupuncture needles connected with HANS. Phase shifts were examined following EA. **(A)** Representative double-plotted actograms of phase shifts. **(B)** Comparison of phase shifts between control *vs*. EA at the same circadian time. Phase advances at CT4 and CT8 in the EA group (CT4 EA group *p* = 0.031, *n* = 6 mice. CT8 EA group *p* = 0.007, *n* = 6 mice) and phase delays at CT16 in the EA group (*p* = 0.022, *n* = 6 mice). One-way ANOVA data are shown as mean ± SEM, *p < 0.05.

Light is the strongest stimulus to regulate the circadian phase (Julius et al., 2019). To avoid potential photic confounds, the experiments were performed in the absence of light cycles to examine the impact of EA on the circadian phase. First, we determined the circadian phase shifts using the wheel running activity. It is a widely used behavioral assay with a stable phenotype (Meijer and Robbers 2014). On day 10 of constant darkness, the EA group mice were treated by EA at CT0, CT4, CT8, CT12, CT16, and CT20, respectively. During DD, the control group showed a free-running rhythm from the phase of the previous light–dark cycle (Figure 2A). EA causes a phase shift, and large phase advances at CT4 and CT8 in the EA group; while phase delays occur at CT16 in the EA group. No differences were observed in phase data among other groups (Figure 2B).

### Influence of EA on Mouse SCN Phosphoproteomic Profile

**FIGURE 3:**
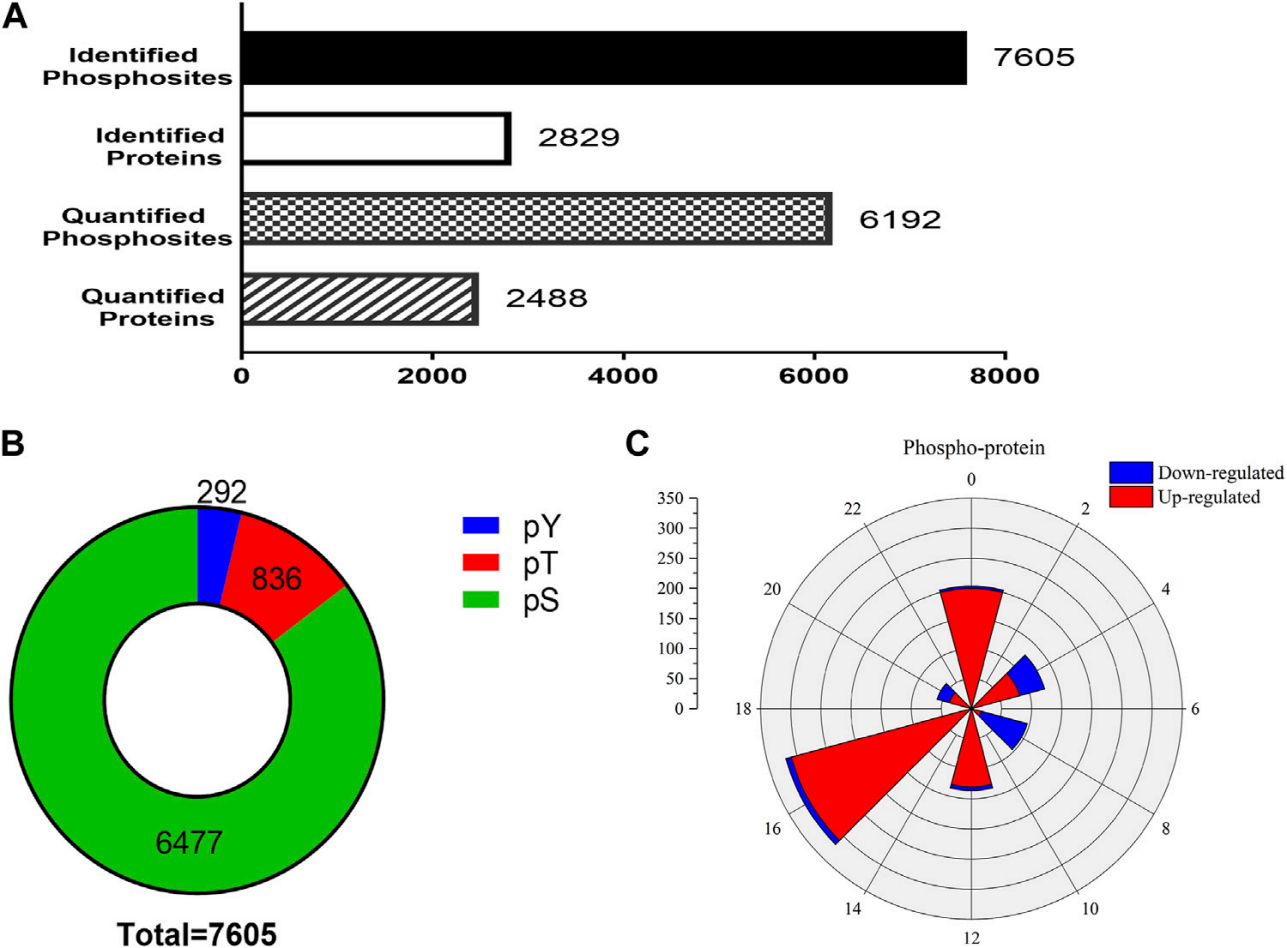
Outline of the phosphoproteomic data. **(A)** The number of identified phosphosites and the quantification of phosphosites and phosphoproteins. **(B)** The proportions of phosphoserine (green), phosphothreonine (red), phosphotyrosine (yellow). The 7,605 phosphosites included 6,477 phosphoserine sites (85.2%), 836 phosphothreonine sites (11%), and 292 pY sites (3.8%). **(C)** Rose plots represent the phase distribution of global analysis for all quantified phosphoproteins. Red, upregulated (>1.2-fold) or blue, downregulated (<1/1.2-fold).

In the phosphoproteomic analysis of the SCN samples, 7,605 phosphosites and 2,829 phosphoproteins were identified (Figure 3A). To ensure the credibility of the results, we used the standard of localization probability >0.75 to filter the identification data (Lundby et al., 2012); consequently, 6,192 distinct phosphosites and 2,488 phosphoproteins were quantified (Figure 3A). Supplementary Material Table S1 summarizes all identified phosphosites. Interestingly, 7,605 phosphosites included 6,477 phosphoserine (pS) sites (85.2%), 836 phosphothreonine (pT) sites (11%), and 292 phosphotyrosine (pY) sites (3.8%), which is consistent with the general pattern of phosphorylation sites in the mouse tissue (Huttlin et al., 2010). We also identified Tpd52 in samples, which were substantiated as a new synaptic protein (Biesemann et al., 2014). These results enabled further high-resolution biochemical analyses.

To check whether EA-induced phase shifts could modify the protein level of SCN, the phosphoproteins that exhibited significant differences between the control and EA groups at the same CT point were selected. Phosphorylation sites with upregulation or downregulation in two biological replicates were arranged. The differences in phosphosites were examined at a 1.2-fold change quantitative ratio as the threshold (Li et al., 2020). The up- and downregulated phosphosites in the EA were compared to the control at different time points. During EA entrainment, the mice showed different resetting patterns of activity initiation between various groups. Interestingly, scanning of the phosphorylation properties in the SCN revealed a significantly different number of proteins grouped into two main segments. We found that most downregulated proteins clustered in CT4 and CT8, while CT16 showed that upregulated proteins were dominant (Figure 3C). These results suggested that EA has significant effects on the phosphorylation pattern of SCN proteins.

### Analysis of Functional Annotations Illustrated that EA Group Proteins Participate in Postsynaptic and Glutamatergic Synapses and have High Levels of Phosphorylation

**FIGURE 4:**
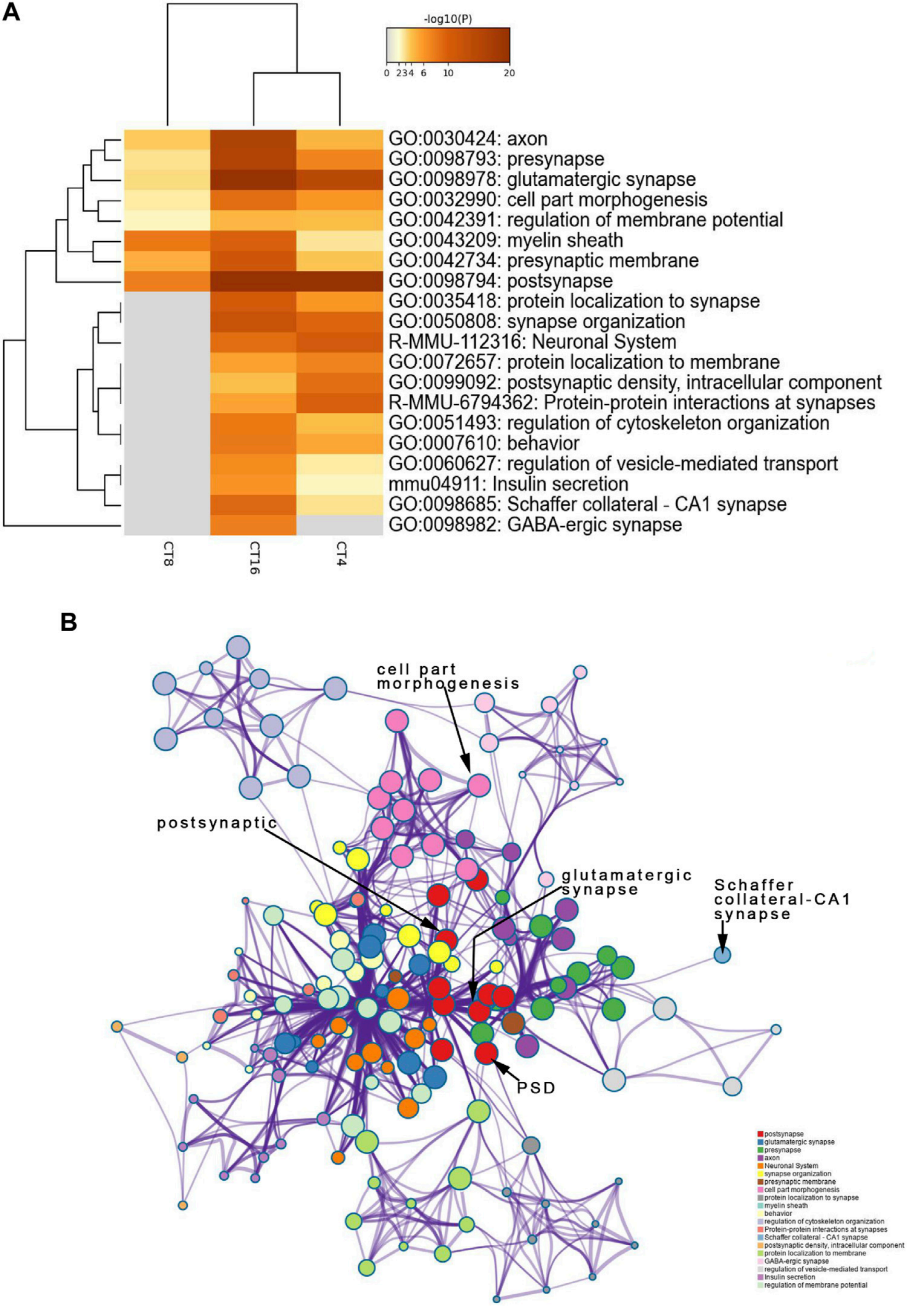
Functional annotation and pathway enrichment analysis of proteins present significantly higher levels in the SCN of the EA group than the mice of the control group. The results are from annotation analysis by Metascape. Metascape selects a subset of representative terms from the full cluster and converts them into a network layout. **(A)** Top 20 items of KEGG pathways, reactome pathway, and GO terms are at a high level in EA. A high -log10 (*p*-value) indicates enrichment. The darker the color, the statistically significant will be the enrichment. **(B)** A higher expression level and an abundance of GO terms in the EA group are represented as networks. Similar terms are organized into groups and colors according to the representative functions of the group. The higher the similarity, the thicker will be the line between the circles. The more similar the function, the tighter will be the connection between the circles. See Supplementary Table S3 for complete list of enriched GO terms.

To identify the SCN-related molecules post-EA, we assessed the unique features defining CT4, CT8, and CT16 groups because CT4, CT8, and CT16 electroacupuncture induced phase shift. Next, the phosphoprotein that exhibited significant differences between the EA and control SCN tissues was subjected to the GO, KEGG, Reactome, and WikiPathways analyses presented in the heat map format. Fisher’s exact test was used to assess CT4, CT8, and CT16 differentially modified proteins based on the quantified outcome. The phosphoproteins upregulated in the SCN of EA mice were enriched in pathways, GO cellular components, GO molecular function, and GO biological process with respect to post- and glutamatergic synapses. Typically, the proteins including Grin2a, Map1a, and Vps35, with >1.5-fold change in the SCN of EA mice, are postsynaptic (Supplementary Material Table S2). Most postsynaptic proteins such as NMDAR, mGluR (Grm1), CAMK2 (Camk2b and Camk2d), Shank (Shank1 and Shank3), SynGAP (Syngap1), and nNOS (Nos1) showed upregulated expression between EA and control. The differentially expressed phosphoproteins focused on postsynaptic plasticity. The NMDA receptor and CAMK2 are strongly implicated in mediating synaptic plasticity (Bayer et al., 2006). CAMK2/NR2B interaction effect is postsynaptic (Barcomb et al., 2016). Only CAMK2*β* has the morphogenic activity that regulates the extension and fine dendrites and the number of synapses (Fink et al., 2003). The phosphorylation of CAMK2*β* in S367 is correlated to the increased phosphorylation of Grin2b, also known as NR2B, which is the substrate of CAMK2 (Omkumar et al., 1996). The phosphorylation level of proteins, associated with Schaffer collateral–CA1 synapse and postsynaptic density (PSD), was also increased in CT4 and CT16. Consistent with the upregulation of proteins involved in excitatory synapses, the long-term potentiation (LTP) was also higher in SCN (Supplementary Material Table S3). The best example of plasticity is LTP, which requires the action of glutamate receptor NMDAR to be enriched in the Schaffer collateral (Lüscher and Malenka 2012). The changes in the PSD of excitatory synapses are essential for LTP (Li et al., 2016). Interestingly, this function of mediating the PPIs at synapses suggested that EA at CT4 and CT16 induces LTP for network clusters (Figure 4B).

### Downregulated Phosphorylation Proteins Involved in Postsynapses, Dendrites, and Neuronal Systems

**FIGURE 5:**
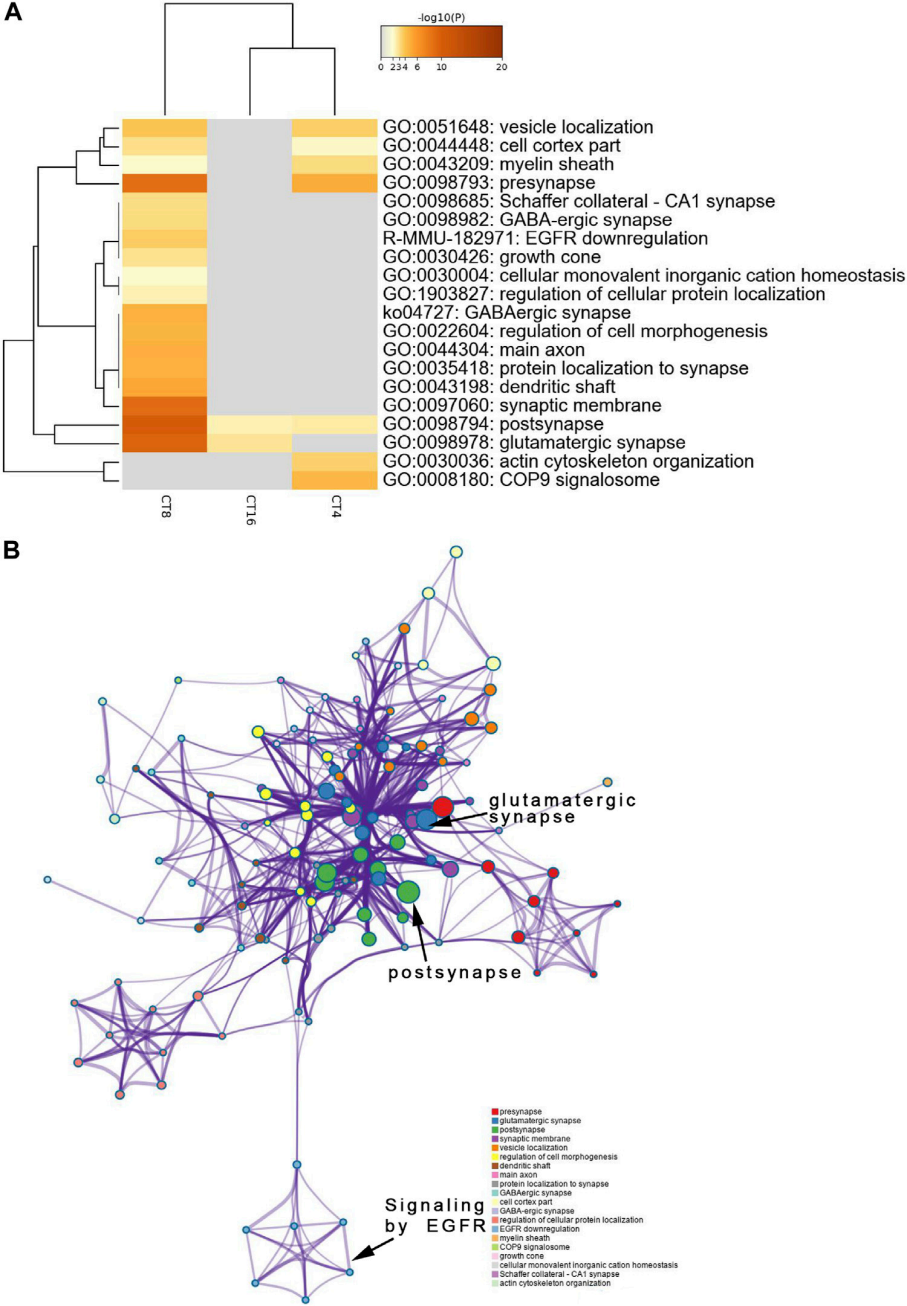
Functional annotation and pathway enrichment analysis of proteins present significantly lower levels in the SCN of the EA group than the mice of the control group. The results are from annotation analysis by Metascape. Metascape selects a subset of representative terms from the full cluster and converts them into a network layout. **(A)** A low expression level and abundance of GO terms in the EA group are represented as networks. Similar terms are organized into groups, and the color is according to the representative functions of the group. The higher the similarity, the thicker will be the line between the circles. The similar the function, the tighter will be the connection between circles. A higher -log10 (*p*-value) indicates more enrichment (Fisher’s exact test). The darker the color, the more statistically significant will be the enrichment. See Supplementary Table S4 for complete list of enriched GO terms.

**FIGURE 6:**
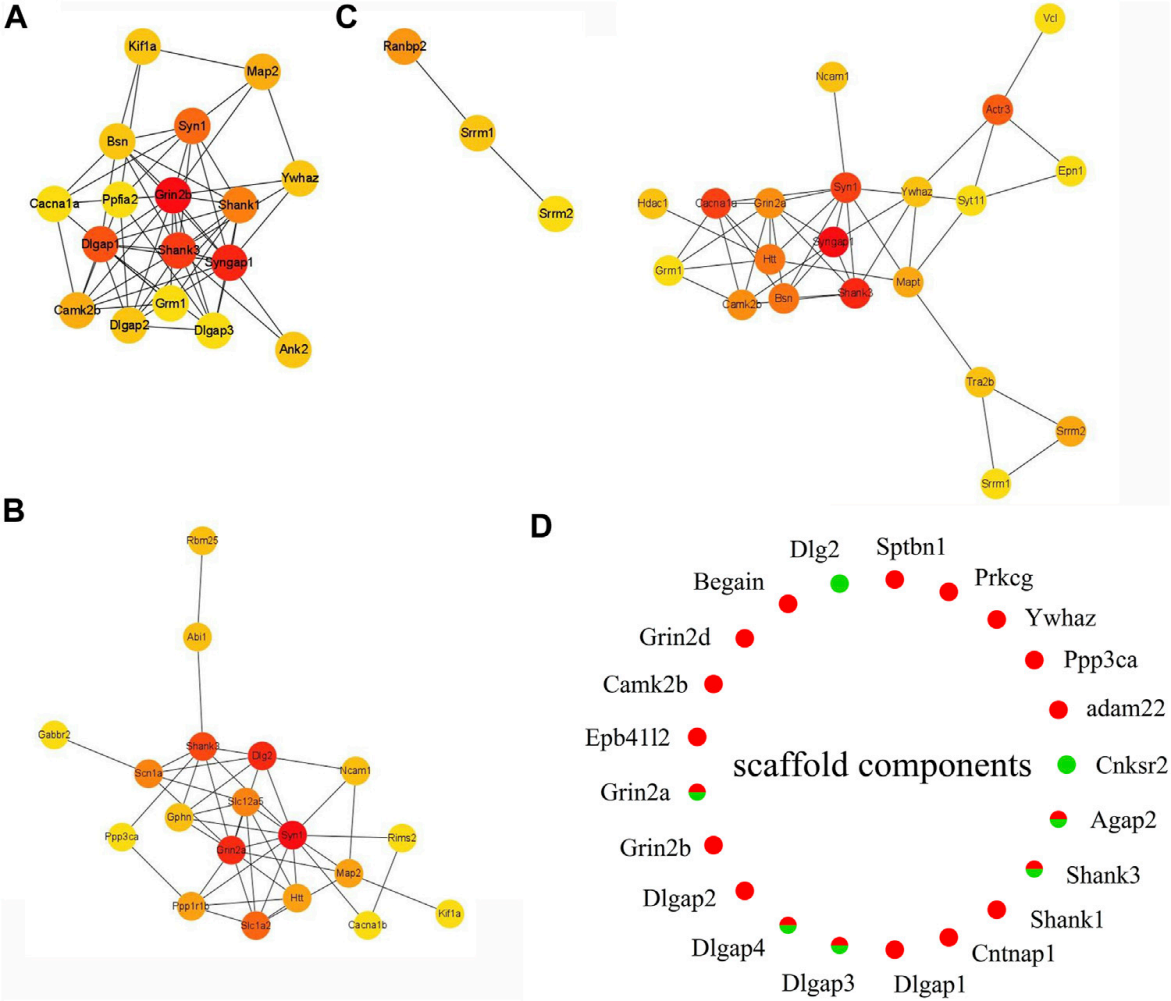
Expression heat map of top 20 hub proteins. **(A)** EA-CT4 *vs* Con-CT4. **(B)** EA-CT8 *vs*. Con-CT8. **(C)** EA-CT16 *vs*. Con-CT16. **(D)** Extracted and assessed upregulation and downregulation status of the phosphorylation of the core scaffold proteins. Red, upregulated (>1.2-fold); green, downregulated (<1/1.2-fold).

The decreased phosphosites were subjected to pathway analyses. Proteins in postsynapse categories including Cnksr2, PCLO, Ccm2, and Ppfia2 were present at > 1.5-fold levels (Supplementary Material Table S2) and known as scaffold proteins (Lim et al., 2014; Miura et al., 2017). The decrease in PCLO corresponds to the downregulation of EGFR in EA compared to the control (Figure 5B). PCLO promotes EGFR-dependent signaling (Zhang et al., 2017). Interestingly, several sites in the CT8 group showed decreased phosphorylation after EA compared to the CT4 and CT16 groups. Glutamatergic synapse was significantly downregulated in CT8, which included Abi1 (S225), Cacna1b (S2221), Grin2a (S929), Ncam1 (T1030), Slc1a2 (S542), Dlg2 (Y364), Shank3 (S995), Rims2 (S965), and Gabbr2 (T762). These proteins exhibited maximal connectivity at CT8 in PPI analysis (Figure 6B). Other proteins at CT8 present at significantly lower levels were involved in the *γ*-aminobutyric acid (GABA)ergic synapse pathway, including Cacna1b, Gng12, Slc12a5, Gabbr2, and Gephyrin. Gephyrin proteins are located in the GABAergic synapse. Moreover, Gephyrin is a major factor that anchors and stabilizes GABA receptors on the synapses of the brain (Choii and Ko 2015). In addition, Slc4a4 was identified in only CT8 at > 1.5-fold lower levels in SCN (Supplementary Material Table S2). Also, S257 phosphorylation of Slc4a4 was crucial for transport activity (Khakipoor et al., 2019). The upregulated and downregulated phosphor-proteins were consistent in many terms in the enrichment analysis such as post- and glutamatergic synapses (Figure 4A and Figure 5A).

PPI analysis shows that the phosphorylation of high–connectivity degree core scaffolding proteins indicate synaptic plasticity. In the PPI networks, the most enriched three-node proteins with high connectivity degree between EA-CT4 and Con-CT4 groups are shown as red nodes (Figure 6A): Grin2b, Syngap1, Shank3, Syngap1, and Neurabin-II (PPP1R9B) (Supplementary Material Table S1) show increased phosphorylation; both play critical roles in synaptic plasticity, LTP, and learning (Siino et al., 2018). SynGAP is abundant in the PSD and is also known to be a synaptic substrate for CAMK2 (Kennedy 2000). Syn1, Grin2a, and Dlg2 are the three most enriched proteins with the highest contact between the EA-CT8 and Con-CT8 groups (Figure 6B). Syngap1, Shank3, and Syn1 exhibited maximal connectivity between the EA-CT16 and Con-CT16 groups (Figure 6C). Synapsin 1 is widely used as a sensitive indicator of synaptogenesis and is the highly connected hub node in PPI networks (Mundy et al., 2008). We found that Synapsin 1 was upregulated in CT4 and CT16 and downregulated in CT8, which is consistent with the results of functional annotation analysis.

According to PPI analysis, structural proteins including cytoskeletal (microtubule-associated protein 2, MAP2), scaffold (Shank1, Shank3, Dlg2, Dlgap1, Dlgap2, Dlgap3, Dlgap4), cell adhesion (Ncam1), and 14-3-3 molecules (Ywhaz) (Figures 6A–C) are common factors (Li, Wilkinson, Clementel, Hou, O'Dell and Coba 2016). DLGs, DLGAPs, SHANKs, and SynGAP1 are considered PSD core scaffold components that interact with various PSD scaffold proteins. To evaluate the changes in PSD, we extracted the phosphorylated scaffold proteins in SCN and marked them in a circle map (Figure 6D). The most identified scaffold proteins (19/21) are upregulated, while five are downregulated (Grin2a, Dlgap3, Dlgap4, Shank3, and Agap2). The phosphorylation levels of Grin2a, Shank3, Dlgap3, Dlgap4, and Dlg2 are decreased at CT8. We also found that the most closely linked proteins in the PPI networks were the core scaffold proteins. Taken together, these data indicated that compared to control, EA is associated with the phosphorylation of core scaffold proteins, and the expression changes are primarily postsynaptic. Furthermore, these phosphoproteins are correlated with synaptic plasticity.

### Validation of TMT-Based Results

**FIGURE 7:**
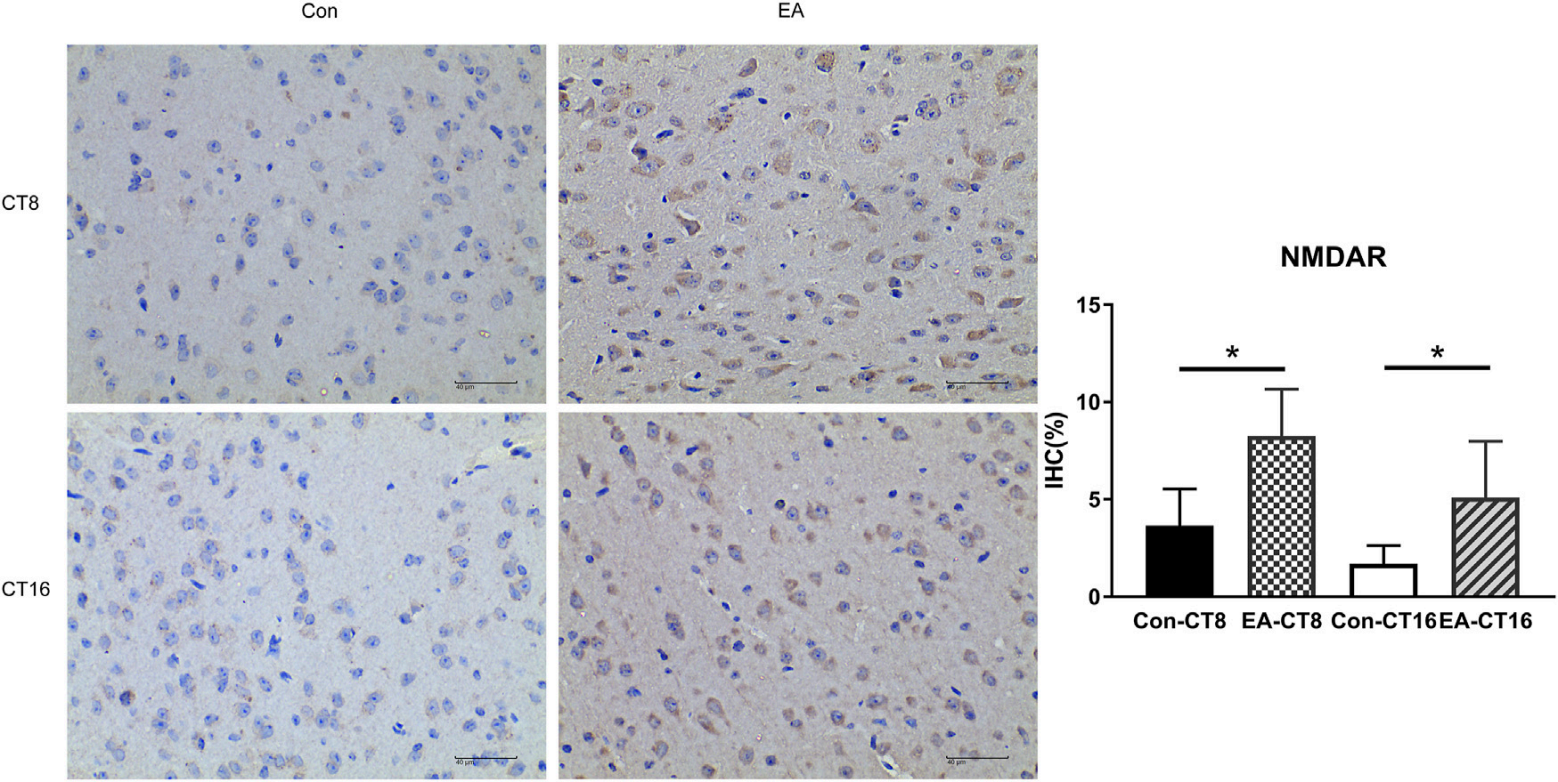
Representative IHC images of NMDAR. All the tissues were observed at ×400. Halo 101 data analysis system was applied to calculate the percentage of positive area of each image. The positive expression was brown. Compared to the control group, the NMDAR in the SCN of the EA group was significantly increased (CT8 EA group *p* = 0.01, CT16 EA group *p* = 0.037); *n* = 5 mice each group, Student’s *t*-test, *p < 0.05.

**FIGURE 8:**
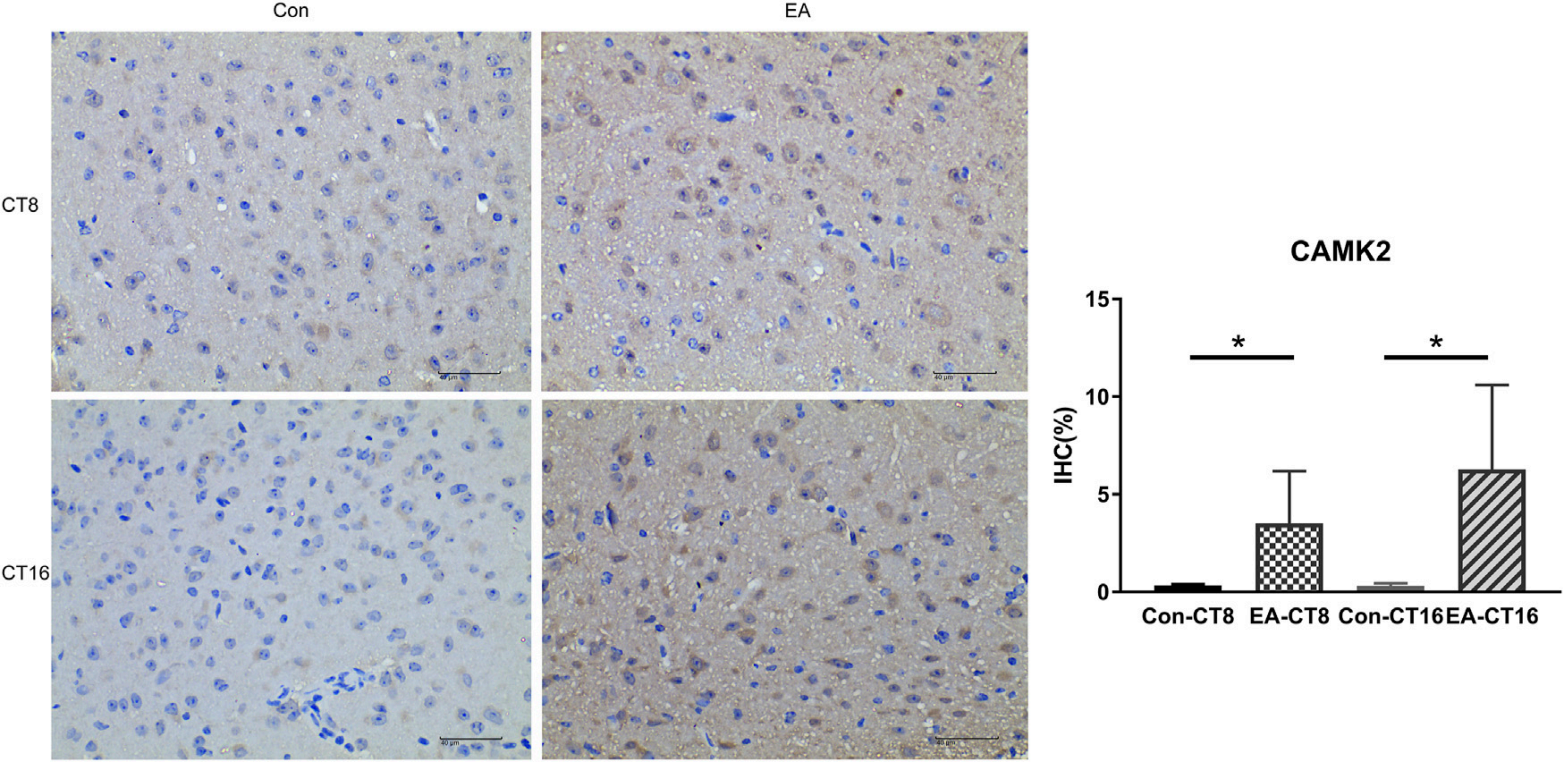
Representative IHC images of CAMK2. All the tissues were observed at ×400. The positive expression was brown. Compared to the control group, the CAMK2 in the SCN of the EA group was significantly increased (CT8 EA group *p* = 0.029, CT16 EA group *p* = 0.036); *n* = 5 mice each group, Student’s *t*-test, *p < 0.05.

The target proteins, NMDAR and CaMK2, were selected for verification using IHC. Compared to the control and the EA groups at the same CT time point, IHC revealed that the changes in NMDAR and CAMK2 were significant. Significant differences were noted in NMDAR (Figure 7) and CAMK2 (Figure 8) between the EA-CT8 and Con-CT8 groups and between EA-CT16 and Con-CT16 groups. IHC also showed that the two candidate proteins had trends similar to those observed in the TMT results, supporting the proteomics data.

## Discussion

To the best of our knowledge, these findings are novel. The current study provides a glimpse at the effects of EA-induced phase shift on the phosphoproteomic patterns in constant darkness in mice SCN. Most studies on proteomics responses to acupuncture have focused on analgesia and neuroprotective mechanisms (Sung et al., 2004; Yang et al., 2018). Only one study has attempted to identify the phosphoproteins of EA in an inflammatory pain model (Lee et al., 2012 Jul). In this study, we investigated the effect of EA at various time points on the characteristics of mice circadian rhythm in the absence of an external light–dark cycle. EA causes phase advances during the subjective day and delays during the subjective night (Figure 2B). Our data demonstrated that EA treatment elicits partial reparation of circadian rhythm exposed to constant darkness. The phase of EA changes shows a bidirectional effect in mice under constant darkness.

Reportedly, EA regulates the expression of circadian rhythm genes (*Per1* and *Per2*) in SCN under the pathological condition of morphine tolerance. However, no differences were observed in the expression levels of *Per1* and *Per2* between the wild-type (WT) control group and EA group (Chen et al., 2017; Hong et al., 2018), so we performed further analysis. Phosphorylation events correspond to phase shifts of circadian rhythms, with both advances and delay (Golombek et al., 2004). Here, phosphoproteomics and PPI analysis showed complicated changes in proteins critical to synaptic integrity and function in the SCN. The comparison of individual phosphor-protein changes at each time point revealed that distinct phosphorylation sites were up- or downregulated in SCN at the different time points (Figure 3C). These data indicated that phosphorylation might be a key mechanism for EA-induced resynchronization patterns. The GO analysis showed that the term “postsynaps” in all CT groups was significantly enriched in cellular components, which might be the general efficacy of EA. Since CT4 and CT8 found that the biological process (BP) term was related to cell morphogenesis, and the BP term of CT16 involved regulation of neurotransmitter transport, and these three unique enrichment terms were not found in other groups without phase shift, which may indicate the specific role of phase regulation in EA. The synaptic molecular functions related to sleep–wake cycles are mainly enriched in glutamatergic and GABAergic synapses (Figure 4A and Figure 5A) (Brüning et al., 2019). Glutamate and GABA levels play a notable role in the adjustment of the sleep–wake cycle in SCN, thereby confirming that the glutamatergic and GABAergic synapse pathways are involved in EA.

Furthermore, we determined that differential phosphorylation of proteins involved in postsynapse in the SCN of EA compared to the control mice. The most enriched functional groups represented structural proteins involved in the regulation of cell morphogenesis and synaptic plasticity (Figure 4A and Figure 5A). PPI analysis showed that the candidate proteins, NR2A, NR2B, Shanks, and Syngap1 exhibited the highest connectivity degree between the EA and control groups. The NMDA receptor and CAMK2 may serve as signal transduction molecules and synaptic structural components in PSD (Chen et al., 1998; Ehlers 1999). PSD plays a critical role during synaptic plasticity. After 2 weeks of acupuncture, PSD was significantly increased, and the synapse cleft width was narrowed in ischemia/reperfusion rats (Xia et al., 2017 Apr). Conversely, our phosphoproteomics data demonstrated that EA entrainment only once is sufficient to trigger changes in the phosphorylation of the component proteins of the postsynaptic density in SCN. Next, we extracted the scaffold proteins in SCN to explore the changes of proteins in PSD. Similar to the results of function annotation analysis, most identified scaffold proteins were upregulated. As evidence for synaptic plasticity, hub proteins are termed “scaffold interactors.”

Although the neural and the molecular basis of mammalian rhythms have been elucidated, the practical tools to rapidly adjust the clock to accommodate shift work and trans-meridian jet travel are still lacking. Reportedly, the effect of EA in the phase shifts would provide the chronotherapy tool. The deficiency of this study lies in that no corresponding dose-related research is carried out. For the parameter setting of EA, it refers to previous research. In future studies, we will focus on dose-related responses to different EA intensity research and the role of glutamatergic and GABAergic synapses and neurotransmitters in EA-induced transmission and synaptic plasticity. Therefore, the current study would provide new insights to clarify the role of these proteins and phosphosites in SCN, especially related to synaptic plasticity.

## Data Availability

The mass spectrometry proteomics data presented in the study are publicly available in ProteomeXchange Consortium (http://proteomecentral.proteomexchange.org) *via* the iProX partner repository with the dataset identifier PXD029450.
