# Supplementary figures and images for: Synaptic Protein Phosphorylation Networks Are Associated With Electroacupuncture-Induced Circadian Control in the Suprachiasmatic Nucleus
Source: Front Genet. 2021 Dec 16;12:762557. doi: 10.3389/fgene.2021.762557 (PMC8717940; doi:10.3389/fgene.2021.762557)

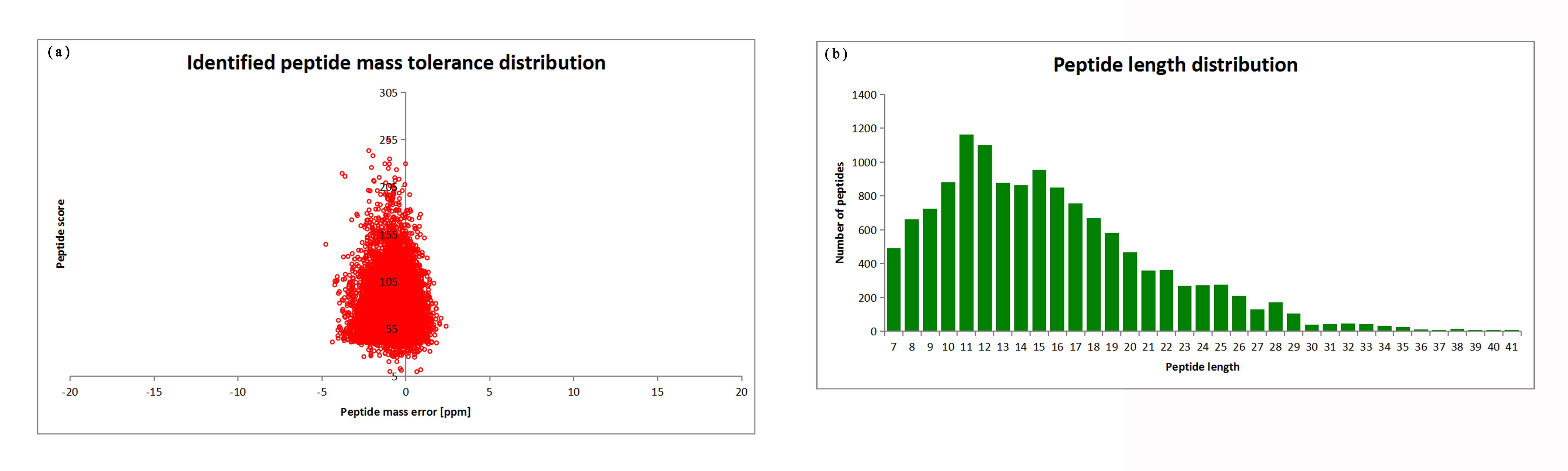

Supplement: Supplementary file 2 [file Image1.TIF]
